# Accumulation of Major Life Events in Childhood and Adult Life and Risk of Type 2 Diabetes Mellitus

**DOI:** 10.1371/journal.pone.0138654

**Published:** 2015-09-22

**Authors:** Jolene Masters Pedersen, Naja Hulvej Rod, Ingelise Andersen, Theis Lange, Gry Poulsen, Eva Prescott, Rikke Lund

**Affiliations:** 1 Section of Social Medicine, Institute of Public Health, University of Copenhagen, Copenhagen, Denmark; 2 Center for Health Aging, University of Copenhagen, Copenhagen, Denmark; 3 Copenhagen Stress Research Center, Copenhagen, Denmark; 4 Section of Biostatistics, University of Copenhagen, Copenhagen, Denmark; 5 Department of Cardiology, Bispebjerg University Hospital, Copenhagen, Denmark; Hunter College, UNITED STATES

## Abstract

**Background:**

The aim of the study was to estimate the effect of the accumulation of major life events (MLE) in childhood and adulthood, in both the private and working domains, on risk of type 2 diabetes mellitus (T2DM). Furthermore, we aimed to test the possible interaction between childhood and adult MLE and to investigate modification of these associations by educational attainment.

**Methods:**

The study was based on 4,761 participants from the Copenhagen City Heart Study free of diabetes at baseline and followed for 10 years. MLE were categorized as 0, 1, 2, 3 or more events. Multivariate logistic regression models adjusted for age, sex, education and family history of diabetes were used to estimate the association between MLE and T2DM.

**Results:**

In childhood, experiencing 3 or more MLE was associated with a 69% higher risk of developing T2DM (Odds Ratio (OR) 1.69; 95% Confidence Interval (CI) 1.60, 3.27). The accumulation of MLE in adult private (p-trend = 0.016) and work life (p-trend = 0.049) was associated with risk of T2DM in a dose response manner. There was no evidence that experiencing MLE in both childhood and adult life was more strongly associated with T2DM than experiencing events at only one time point. There was some evidence that being simultaneously exposed to childhood MLE and short education (OR 2.28; 95% C.I. 1.45, 3.59) and work MLE and short education (OR 2.86; 95% C.I. 1.62, 5.03) was associated with higher risk of T2DM, as the joint effects were greater than the sum of their individual effects.

**Conclusions:**

Findings from this study suggest that the accumulation of MLE in childhood, private adult life and work life, respectively, are risk factors for developing T2DM.

## Introduction

Experiencing major life events (MLE), such as divorce or bereavement, is relatively common during a normal life course and many MLE have stressful effects [1–4]. A growing body of evidence suggests that stress is an independent risk factor for type 2 diabetes mellitus (T2DM)[5], and biological evidence shows that both acute and prolonged stressors can result in a disrupted central regulatory system which is associated with visceral obesity and insulin resistance [6,7]. In a similar vein, depression and anxiety, which have been clearly linked with MLE [8,9], are associated with dyslipidemia, increased sympathetic nervous system activity and inflammatory cytokines resulting in visceral obesity, insulin resistance and T2DM [10].

Previous studies addressing the effects of MLE on T2DM have been mixed, depending on the nature and timing of the MLE experienced. When considering MLE in childhood, several studies have assessed the effects of events such as child abuse, neglect and being placed in foster care on T2DM with mixed results ranging from no effect to an over two-fold risk of T2DM [5,11–15]. There is also mixed evidence on the effects of MLE in adult life on T2DM [1,15–18]. A handful of studies have explored the relationship between the accumulation of MLE and T2DM, and findings from these studies have been more consistent [12,16,18]. For example, a cross sectional study found that number of stressful events in adult life was positively associated with the prevalence of hitherto undetected T2DM [16]. An American population based survey suggests that exposure to injurious traumatic events or witnessing traumatic events at unspecified times in life modestly increases risk of T2DM in an exposure dependent manner [18] and among women, physical and sexual abuse in childhood was also found to be associated with risk of T2DM in a dose response manner [12].

The idea that the effect of a given stressor has a differential effect across social economic position is supported by the theory of differential vulnerability [19,20]. According to the theory, there are underlying differences between social groups in their vulnerability to a risk factor (for example MLE) [19]. The increased vulnerability is explained by the lower levels of social and intrapsychic resources as well as higher levels of adverse health behaviours in the lower social groups [20]. This makes the effect of exposure to an additional risk factor potentially more severe among those with a shorter education than among those with a higher education.

Previous studies of the MLE and T2DM association have almost exclusively focused on childhood [11–15] or more commonly adult MLE exposures [1,16,17]. Measures of MLE that occur over the duration of a life are likely to give a fuller picture of the stress an individual has been exposed to. Evidence suggests that most individuals can successfully cope with a stressful life event, however, problems arise when stressors accumulate [21]. Therefore we hypothesize that the accumulation of events in childhood, adult private and adult work life will be associated with higher risk of developing T2DM in an exposure dependent manner. Further, we hypothesize that experiencing MLE in both childhood and adult life will result in a higher risk of T2DM compared with only experiencing MLE at one time period. Due to plausible socioeconomic differences in coping with stressful MLE [20], we hypothesize that the effects of MLE on T2DM will be modified by educational attainment, with the lower educated group being more vulnerable.

## Materials and Methods

The Copenhagen City Heart Study (CCHS) is a longitudinal study initiated in 1976 comprised of an age-stratified random sample from the Copenhagen area. There were 19,698 men and women 20–93 years of age invited to participate in the first wave of the study. A total of 14,223 individuals attended the first examination in 1976–78 (74%). At subsequent waves of the study in 1981–83, 1991–93 and 2001–03 the original sample and a number of new individuals were invited to participate. The CCHS was approved by the Committee of Biomedical Research Ethics for the capital region of Denmark. All participants gave informed written consent. The present study included information on MLE from the third wave of the study (n = 10,135, response rate 61%) which took place from 1991–93, when the population was supplemented with 2,360 men and women aged 20–49. The current study was based on persons with continued participation in the 3^rd^ and 4^th^ waves of the study (n = 5,008). This constitutes a 49% participation rate (22% died between waves 3 and 4 and 29% choose not to participate). The participants included in this analysis were younger, less educated, and less likely to be women than the entire cohort from the third wave of the study, however, there were not significant differences in exposure to MLEor family history of diabetes (data not shown). Participants previously hospitalized with diabetes (n = 4), self-reported diabetes at baseline (n = 89), with non-fasting glucose levels above 200 mg/dl (n = 1), with missing information on MLE (n = 49) or covariates (n = 104) were excluded leaving 4,761 participants for the analysis. The participants excluded due to missing data were on average 1 year younger, less likely to be female, more likely to die over follow up and were more educated than the participants who were included (data not shown). However these differences were not substantial and therefore we do not expect the exclusion of these persons to have severely biased the findings.

### Major Life Events

At wave three (1991–1993) of CCHS, participants filled out a self-administered questionnaire with 18 dichotomous questions about MLE based on a shorter modified version of the Social Readjustment Rating Scale [22]. In accordance with the approach employed by Andersen et al. [23] the MLE were grouped according to the time point (in childhood or adult life) and realm of the life course (adult private or work life) in which they occurred. The participants were asked if they experienced any of the following events during their life:

Childhood MLE: long-term illness in parents, being placed in care outside of the home, serious family conflicts, parents’ long-term unemployment, and parent’s serious economic problems.

Adult MLE: serious or long-term illness in children, long-term illness or serious accident, having children with educational problems, serious conflicts with adult children, marital problems, death or long-term illness in a close family member, and serious economic problems.

Work MLE: job loss, serious conflicts with colleagues, serious conflicts with supervisors, serious conflicts with employees, not being promoted, and not achieving educational goals.

We did not necessarily expect a linear association between number of MLE and T2DM, as e.g. a threshold effect would be equally reasonable to assume. For this reason we grouped the major life events as 0, 1, 2, ≥3 allowing for different patterns to emerge. To test for a dose response association, the MLE were included in the model as a continuous variable (0, 1, 2, ≥3 MLEs) and the corresponding p-values were reported. To examine the effects on T2DM of the combination of MLE and educational attainment new composite variables were created for each category of MLE (childhood, adult private and work life) and education: (1) ≤1 MLE and >9 years education (reference group); (2) ≤1 MLE and ≤9 years education; (3)>1 MLE and >9 years education;(4) >1 MLE and ≤9 years education. Similarly the interaction between MLE in childhood and adult life was categorized as follows: experiencing 0 MLE, only experiencing ≥1 childhood MLE, only experiencing ≥1 adult MLE (private and/or work), experiencing ≥1 childhood and ≥1 adult MLE.

### Type 2 diabetes mellitus

Incident T2DM was defined by self-reported diabetes at wave 4, clinical examination at wave 4 or hospital admission for diabetes. Information on self-reported diabetes was obtained from a questionnaire item (Do you have diabetes?) administered at wave 4 (2000–2003) of the CCHS. Using civil registration links all participants were followed between waves 3 and 4 of the CCHS in the Danish National Patient Register (NPR) which contains information on all hospital admissions in Denmark. All first time hospital admissions (primary or secondary), outpatient activities and emergency room contacts with T2DM were identified using the International Classification of Disease codes (until 1994 ICD 8: 250, 1994 onward ICD 10: E11, E13, E14). Glucose levels were measured by a non-fasting venous blood sample drawn at clinical examination at wave 4 of the CCHS. Non-fasting glucose levels >200mg/dl were considered as a diagnosis of diabetes according to the American Diabetes Association guidelines [24].

### Covariates

Covariates were measured at baseline in 1991–1993 from self-reported questionnaire and included age (continuous), sex, family history of diabetes (either mother or father had diabetes),and educational attainment (≤9 years, > 9 years) with 9 years of education corresponding to mandatory education among people born after 1959 in Denmark. Confounders were identified based on a prior knowledge and according to the directed acyclic graph method [25].(S1 Fig)

### Statistical Methods

Multivariable logistic regression models were used to estimate the association between MLE and T2DM. The first model was unadjusted, the second adjusted for age, sex, family history of diabetes and education. The potential modification of the accumulation of MLE in childhood, private adult and work life and T2DM by educational status was assessed by estimating the joint effect of exposure to MLE in each domain and educational status. In accordance with the STROBE recommendations [26], the separate effects of education and MLE and their joint effects with one reference category are presented. Similarly, to test the hypothesis that the accumulation of childhood and adult MLE would incur a greater risk of T2DM than only experiencing MLE at one time point, we modeled the joint effect of the exposures. The Relative Excess Risk Due to Interaction (RERI) with 95% C.I. based on Wald-type statistics using approximate variance estimators are presented with the joint effect estimates [27]. To shed light on the prevalence and association of individual MLE on risk of T2DM, a sensitivity analysis was conducted to estimate the association between individual MLE and risk of T2DM controlling for age, sex, family history of diabetes and education. All analysis were performed in Stata version 12.0 and p-values were 2 sided.

## Results

During ten years of follow up 198 (4%) of participants developed T2DM. There were 180 self-reported cases, an additional 13 cases identified in the registries and 5 cases identified by non-fasting glucose samples. The mean age at baseline was 53 years (range 21–87). Approximately 45% of the participants experienced MLE in both childhood and adult life, whereas 15% of the participants reported not experiencing any MLE (Table 1). Participants were least likely to experience only MLE in childhood (7%). Participants who experienced both childhood and adult events were younger (mean age 52) and more likely to be women (62%). There were differences in education level, with those who only experienced MLE in adult life or in both adult and work life being less educated than the zero MLE and only childhood MLE groups.

**Table 1:** Baseline Characteristics of 4,694 participants of the third and fourth waves of the Copenhagen City Heart Study by experience of major life events.

	Zero MLE (N = 725)	Only child MLE (N = 332)	Only adult MLE (N = 1,581)	Both child & adult MLE (N = 2,123)	P-value*
**Diabetes cases; %**	3	5	4	4	0.546
**Age; mean**	54	54	53	52	<0.000
**Women; %**	51	53	59	62	<0.000
**Education <9 years; %**	51	50	43	46	0.003
**Family history of diabetes; %**	10	10	11	12	0.169

*P-values are from chi-squared test *tests or ANOVA*.

### Childhood

Experiencing 3 or more childhood MLE was associated with a 69% (Odds ratio (OR) 1.69; 95% confidence interval (C.I.) 1.06, 2.70) higher risk of developing T2DM compared with not experiencing childhood events, but there was no clear evidence of a dose response association (p = 0.09) (Table 2). After adjustment for age, sex, education and family history of diabetes the associations persisted. In childhood, long term illness in parents was the most prevalent MLE with 33% of participants reporting the event. Long term illness in parents was associated with a 34% higher risk of T2DM (95% C.I. 0.99, 1.81), but there was no evidence of higher risk of T2DM associated with any other single event in childhood (S1 Table).

**Table 2:** Risk of incident type 2 diabetes mellitus among 4,761 men and women by number of major life events (MLE) in childhood, adult private life and work life Odds ratios (OR) and ±95% confidence intervals (CI) are presented.

		Type 2 diabetes mellitus	
	No. of cases/total	Crude OR (95% CI)	Multiple Adjusted OR* (95% CI)
**Childhood MLE**			
0	92/2,306	Reference	Reference
1	58/1,427	1.02(0.73,1.43)	1.07(0.76,1.50)
2	23/638	0.90(0.56,1.43)	1.02(0.64,1.64)
3+	25/390	1.65(1.05,2.60)	1.69(1.06,2.70)
*P*_*trend*_		0.173	0.086
**Adult Private MLE**			
0	52/1,405	Reference	Reference
1	65/1,648	1.07(0.74,1.55)	1.20(0.82,1.76)
2	46/1,025	1.22(0.82,1.83)	1.39(0.92,2.10)
3+	35/638	1.41(0.91,2.18)	1.70(1.08,2.66)
*P*_*trend*_		0.102	0.016
**Work life MLE**			
0	119/2,968	Reference	Reference
1	53/1,197	1.07(0.76,1.9)	1.15(0.81,1.62)
2	17/401	1.06(0.63,1.78)	1.30(0.76,2.22)
3+	11/195	1.43(0.76,2.70)	1.92(1.00,3.73)
*P*_*trend*_		0.348	0.049

*Multiple adjusted OR are adjusted for age, sex, family history of diabetes and educational attainment.

### Adult life: Private and work

There was a dose response relationship in the association between experiencing adult private MLE and developing T2DM, (p-trend = 0.016) and participants who experienced 3 or more MLE in the adult private domain had a 70% higher risk of T2DM (OR 1.70; 95% C.I. 1.08, 2.66). There was also a dose response relationship between work life MLE and T2DM (p trend = 0.049) with experiencing 3 or MLE in work life being associated with a 92% higher risk of T2DM (OR 1.92, 95% C.I. 1.00, 3.73). Adjusting the models for age, sex, family history of diabetes and education strengthened the estimates (Table 2). Fifty two per cent of participants reported experiencing the death or long term illness in a close family member making it the most common MLE measured in the current study. In adult private life, the death or long term illness of a close family member (OR 1.37, 95% CI 1.02, 1.84) and serious economic problems (OR 1.53, 95% CI 1.00; 2.34) were associated with a higher risk of T2DM and in work life job loss, reported by 19% of participants (OR 1.67; 95%C.I. 1.18, 2.37) was associated with T2DM (S1 Table).

### Joint effect of childhood and adult life events

The joint effect of experiencing MLE in childhood and adult life (work and/or private) (OR 1.67; 95% C.I. 1.04, 2.68) was less than the expected combination of only experiencing MLE in childhood (OR 1.55; 95% C.I. 0.79, 3.03) or only in adult life (OR 1.60; 95% C.I. 0.94, 0.98, 2.60), (RERI -0.48; 95% C.I. -1.60.67). (Table 3)

**Table 3:** The joint association of child and adult (both work and private life) MLE and risk of type 2 DM. Odds ratios (OR)(±95% confidence intervals) (CI) are adjusted for age, sex, family history of diabetes and education.

	Cases/total	OR (95%CI)
**0 MLE**	23/725	Reference
**Only child MLE**	15/332	1.55(0.79,3.03)
**Only adult MLE**	69/1,581	1.60(0.98,2.60)
**Child & Adult MLE**	91/2,123	1.67(1.04,2.68)

Only child MLE = ≥1 MLE in childhood, Only adult MLE ≥1 MLE in private adult and/or work life, child and adult MLE≥1 in childhood and ≥1 in adult private and or work life.

### Modification by education

Participants simultaneously exposed to many childhood MLE and short education had over a two-fold risk (OR 2.28; 95% C.I. 1.45, 3.59) of developing T2DM over follow up, compared to the unexposed group. This risk exceeds the sum of the individual risks of exposure to few childhood MLE/high education (OR 1.04, 95% C.I. 0.56, 1.94) and short education/few MLE (OR 1.67, 95% C.I. 1.16–2.41) (RERI 0.57; 95% C.I. -0.48,1.61). Similarly the combined effect of being exposed to short education and many work life MLE (OR 2.86; 95% C.I. 1.62, 5.03) exceeds the sum of the individual risks of short education/few MLE (OR 1.71; 95% C.I. 1.21, 2.42) and many work life MLE/ long education (OR 1.12; 95% C.I. 0.56, 2.24) (RERI 1.03; 95% C.I. -0.58,2.63). The OR in the group exposed to many MLE in adult private life and low educational attainment (OR 2.55; 95% C.I. 1.60, 4.05) was less than the sum of the risk of the participants exposed to short education/few MLE (OR 2.16; 95% C.I. 1.41, 3.33) or many MLE/ long education (OR 1.81; 95% C.I. 1.09, 3.01) (RERI -0.43; 95% C.I. -1.64,0.78)(Table 4).

**Table 4:** Odds ratios(OR) and ±95% confidence interval (CI) for the joint association of major life events (MLE) in childhood, adult private life and work life and education on risk of type 2 diabetes mellitus (N = 4,693). Odds ratios adjusted for sex, age, family history of diabetes. The main effects of education are additionally for MLE in childhood, adult private or work life and the main effects of MLE are additionally adjusted for education.

	Cases/Total	OR (95% CI)
**Childhood**		
**Main effects**		
Childhood MLE ≤1	150/3,733	Reference
Childhood MLE>1	48/1,028	1.25(0.89,1.76)
High Education	63/2,581	Reference
Short education	135/2,180	1.78(1.28,2.46)
**Joint effects**		
Few MLE & high education	50/2,038	Reference
Few MLE & short education	100/1,695	1.67 (1.16–2.41)
Many MLE & high education	13/543	1.04(0.56,1.94)
Many MLE & short education	35/485	2.28(1.45,3.59)
**Adult Private**		
**Main effects**		
Adult private MLE ≤1	117/3053	Reference
Adult private MLE>1	81/1,708	1.36(1.01,1.83)
High Education	63/2,581	Reference
Short education	135/2,180	1.80(1.30,2.48)
**Joint effects**		
Few MLE & high education	32/1,648	Reference
Few MLE & short education	85/1,405	2.16(1.41,3.33)
Many MLE & high education	31/933	1.81(1.09,3.01)
Many MLE & short education	50/775	2.55(1.60,4.05)
**Work**		
**Main effects**		
Work MLE ≤1	170/4,165	Reference
Work MLE>1	28/596	1.43(0.93,2.18)
High Education	63/2,581	Reference
Short education	135/2,180	1.82(1.31,2.51)
**Joint effects**		
Few MLE & high education	53/2,187	Reference
Few MLE & short education	117/1978	1.71(1.21,2.42)
Many MLE &high education	10/394	1.12(0.56,2.24)
Many MLE & short education	18/202	2.86(1.62,5.03)

Legend: Few MLE ≤1, Many MLE>1; High education>9 years, Short education≤9 years.

## Discussion

Findings from this study suggest that the accumulation of MLE in childhood, adult private and work, respectively, are risk factors for developing T2DM with evidence of a dose response relationship in adult private and work life domains. Contrary to expectation we did not find evidence to support the hypothesis of interaction between childhood and adult MLE on risk of T2DM. There was some evidence to support the differential vulnerability hypothesis in the childhood and work domains, but not in the private adult domain.

### Comparisons with other studies

Evidence suggests that adverse childhood experiences are associated with changes in biological systems, including the endocrine and immune systems and that these changes can exert long term effects on later health [28]. Our results suggest that the accumulation of three or more childhood MLE resulted in a 69% higher risk of developing T2DM over follow-up, however experiencing only 1 or 2 events was not associated with higher risk, suggesting a possible threshold effect. A study from 2004 showed that childhood neglect was associated with a two-fold higher risk of T2DM, but childhood physical and sexual abuse were not associated with T2DM[14]. Findings from the 1958 British Birth Cohort suggest that childhood adversities (e.g., physical abuse) tended to be associated with T2DM, but in most cases associations were explained by socioeconomic factors. The MLE in childhood measured in the current study, for example experiencing family conflicts, are serious but perhaps more common than MLE in the studies summarized above. Twenty–two per cent of participants reported experiencing at least 2 MLE in childhood. This suggests that accumulation of three or more rather common negative events in childhood may be associated with a higher risk of T2DM in later life.

Similar to our findings, a Swedish cross sectional study found that the number of stressful life events in adulthood was positively associated with the onset of T2DM. Evidence from the Whitehall II study showed that the accumulation of adult MLE was a moderate but insignificant risk factor for developing T2DM [1]. Further, an American population based survey suggests that exposure to traumatic events occurring anytime during life modestly increases risk of T2DM in an exposure dependent manner [18]. Our study found a dose response trend in exposure to MLE in adult private life and the workplace. We were able to expand on previous findings by addressing childhood and adult MLE separately and estimating if exposure to MLE at both time points was more detrimental than only experiencing the events in one domain. Contrary to expectation, our study did not support the hypothesis of a positive interaction between childhood and adult MLE. However, the MLE included in this study were by no means exhaustive, for example childhood abuse and parental death were not a part of the scale, and we cannot rule out the possibility of an interaction between the childhood and adult domain when considering other MLE. Furthermore, due to the sample size in the current study, the RERI should be interpreted with caution.

To our knowledge, no previous studies have investigated the combined effect of educational attainment and MLE on risk of developing T2DM. We found some evidence that persons with a shorter education were more vulnerable to the effect of MLE in childhood and work on T2DM than their counterparts with more education. Research shows that persons with lower social position are more emotionally affected by negative life events than those with higher status[20]. This is in part due to a greater access to coping resources, including personality characteristics[20], comparatively limited access to supportive social relationships [29], and disadvantaged access to resources such as control over the environment which has been shown to be positively associated with class [20,29]. This could also apply to coping with the lasting effects of exposure to MLE in childhood. The modification of the effect of MLE at work by education could be due to the nature of the work place and conditions of employment associated with jobs available to persons with a low level of education. For example, Kumari et. al showed that men who experienced effort reward imbalance at work had a 60% higher risk of developing T2DM than men who didn’t experience this imbalance [3]. In this study, education was not found to moderate the effect of private adult MLE on T2DM possibly due to the nature of the MLE measured.

### Mechanisms

A behavioral pathway linking stress with T2DM, in which emotional stress is associated with detrimental life style behaviors such as excessive alcohol consumption, overweight, physical inactivity, and tobacco smoking, was suggested in the CCHS [30]. These lifestyle factors are most likely situated on the causal pathway between MLE and T2DM, and are therefore possible mediators of the association.

### Strengths and limitations

The prospective design of CCHS and the exclusion of participants with T2DM at baseline ensured temporality between MLE and developing T2DM. The study additionally included comprehensive data on common yet serious MLE in childhood and different domains of adult life. Moreover, this study compliments the existing literature by addressing the joint effect of childhood and adult MLE on T2DM and by evaluating if this association is modified by education.

The MLE in the study were reported in retrospect, however recall bias is quite unlikely due to the prospective design of the study. Further, the MLE scale did not include time frames making it difficult to ascertain the time lapse between exposure and event. Misclassification of T2DM is also of concern due to the unspecific nature of the question participants could be reporting either type 1 or 2 diabetes. However, type 1 diabetes is usually diagnosed in children and young adults and only 5–10% of people with diabetes have type 1[31]. The mean age of participants at baseline was 52 years, thus this is unlikely to be a major source of misclassification in the current study. Further, the majority of cases of T2DM were self-reported. Incident self-reported T2DM has been found to be a reliable measure of diabetes when compared with general practitioner reports [32]. However in cases of undetected T2DM the study was strengthened by the inclusion of non-fasting glucose measures from the CCHS clinical examinations and data from the National Patient Register.

Residual confounding by socioeconomic status of the association between MLE and T2DM is of concern. Educational attainment was categorized into two broad categories (≤9 years, >9 years) in accordance with mandatory educational requirements in Denmark, to facilitate sufficient statistical power in the joint effects estimates. A remarkable amount of evidence suggests a social gradient in health running from the top to the bottom of society, with poorer health each step down[33], and due to the dichotomization we were unable to address this gradient. Further, SEP is multidimensional and aspects of social position such as status were not adjusted for. Finally, the meaning of education changes over time[34] and due to the considerable broad age range in the study, it is possible that the modification of the association between MLE and T2DM varied by birth cohort or period, however in the current study we did not have the sufficient statistical power to address this potential modification.

## Conclusion

In conclusion, this study finds that the accumulation of MLE in childhood, adult private, and work life is associated with developing T2DM over a ten year period. We did not find evidence of an interaction between childhood and adult MLE leading to an excessive risk of T2DM, contrary to our original hypothesis. Further, in the childhood and work domains, we found some evidence of differential vulnerability by educational status to the effects of MLE on T2DM. Future studies should aim to clarify the effects and explore potential explanatory mechanisms leading from MLE to T2DM.

## Supporting Information

S1 Fig Simplified causal diagram for relation between major life events and type 2 diabetes mellitus. (TIF)

S1 Table Risk of incident type 2 diabetes mellitus (T2DM) by individual major life events in childhood, adult life and work life. Odds ratio (OR) are adjusted for age, sex, education and family history of T2DM.
(DOCX)

## Abbreviations

T2DM: Type 2 diabetes mellitus
CCHS: Copenhagen City Heart Study
MLE: Major life events
