# Supplementary figures and images for: Accumulation of Major Life Events in Childhood and Adult Life and Risk of Type 2 Diabetes Mellitus
Source: PLoS One. 2015 Sep 22;10(9):e0138654. doi: 10.1371/journal.pone.0138654 (PMC4578856; doi:10.1371/journal.pone.0138654)

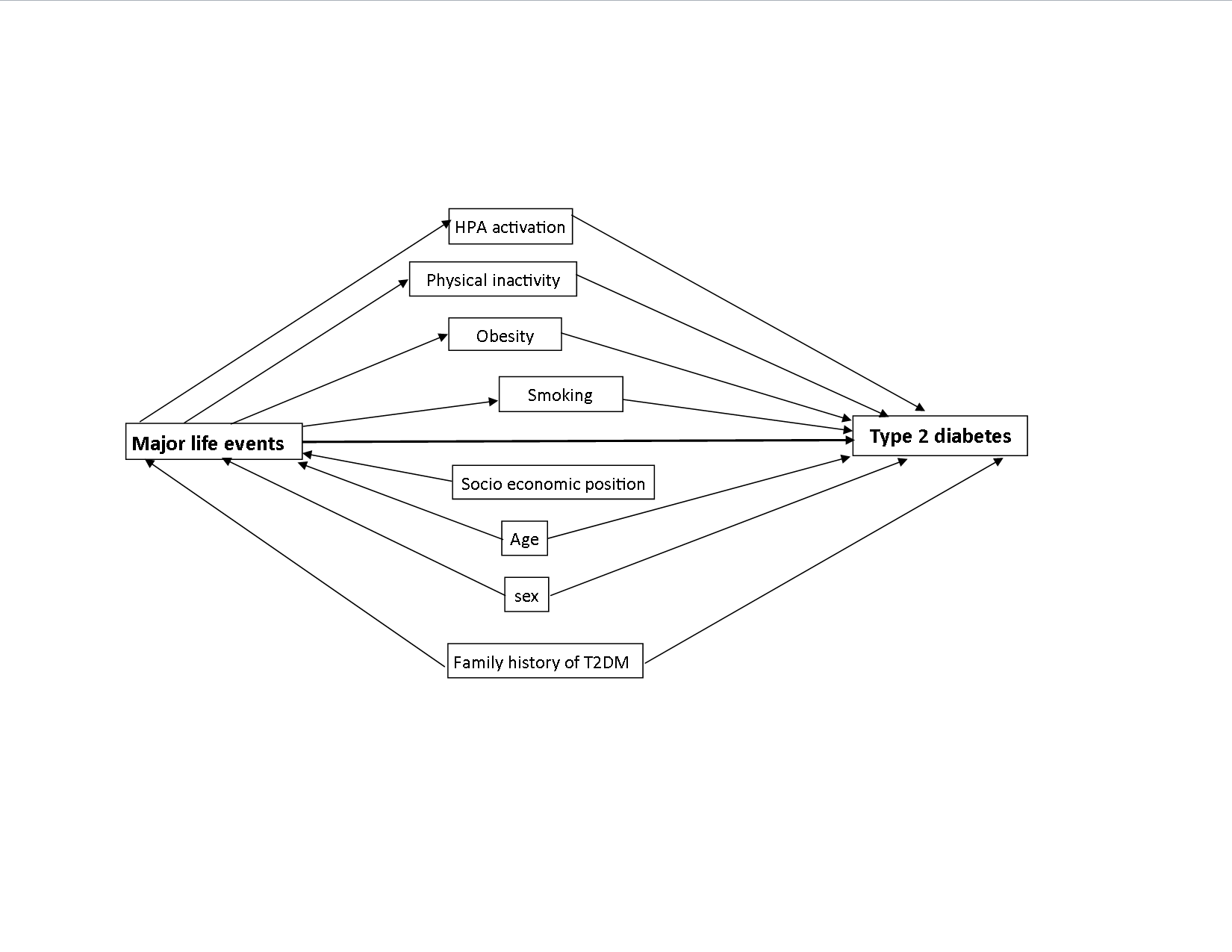

Supplement: S1 Fig — (TIF) [file pone.0138654.s001.tif]
