# Supplementary material for: Accumulation of Major Life Events in Childhood and Adult Life and Risk of Type 2 Diabetes Mellitus
Source: PLoS One. 2015 Sep 22;10(9):e0138654. doi: 10.1371/journal.pone.0138654 (PMC4578856; doi:10.1371/journal.pone.0138654)
Supplement: S1 Table — Odds ratio (OR) are adjusted for age, sex, education and family history of T2DM. (DOCX) [file pone.0138654.s002.docx]

**Supplementary table 1.** Risk of incident type 2 diabetes mellitus (T2DM) by individual major life events in childhood, private adult and work life. Odds ratio are adjusted for age, sex, education and family history of T2DM.

|  | (%) reporting event | Cases/total | Odds Ratio (95 % CI) |
| --- | --- | --- | --- |
| Childhood |  |  |  |
| Long-term illness in parents | 33 | 77/1,548 | 1.34 (0.99; 1.80) |
| Placed in care outside of the home | 9 | 25/546 | 1.21 (0.78; 1.87) |
| Serious family conflicts | 19 | 29/892 | 1.00 (0.66; 1.50) |
| Parents’ long-term unemployment | 7 | 19/336 | 1.14 (0.69; 1.86) |
| Serious economic problems | 15 | 33/697 | 1.11 (0.75; 1.63) |
| Adult private |  |  |  |
| Serious or long-term illness in children | 11 | 21/502 | 0.91(0.57; 1.46) |
| Having children with major educational problems | 6 | 10/281 | 0.83 (0.43;1.60) |
| Major conflicts with adult children | 6 | 16/273 | 1.31 (0.77; 2.24) |
| Marital problems | 15 | 28/713 | 0.83 (0.54; 1.28) |
| Involved in serious accident or long-term illness | 10 | 34/495 | 1.01 (0.70; 1.45) |
| Death or long-term illness in a close family member | 52 | 112/2,454 | 1.37 (1.02; 1.84) |
| Serious economic problems | 4 | 170/198 | 1.53 (1.00; 2.34) |
| Work life |  |  |  |
| Not achieving educational goals | 16 | 39/763 | 1.35 (0.93; 1.95) |
| Job loss | 19 | 49/884 | 1.67 (1.18; 2.37) |
| Not being promoted | 5 | 11/224 | 1.11 (0.59; 2.09) |
| Serious conflicts with colleagues | 6 | 7/291 | 0.78 (0.36; 1.69) |
| Serious conflicts with supervisors | 8 | 15/377 | 1.24 (0.71; 2.16) |
| Serious conflicts with charges | 2 | 4/115 | 1.06 (0.38; 2.94) |
